# Antinociceptive effect of PnTx4(5-5), a peptide from *Phoneutria nigriventer* spider venom, in rat models and the involvement of glutamatergic system

**DOI:** 10.1590/1678-9199-JVATITD-2019-0022

**Published:** 2019-08-12

**Authors:** Camila Franco Batista Oliveira, Daniela Pereira Alves, Bruna Luiza Emerich, Suely Gomes de Figueiredo, Marta do Nascimento Cordeiro, Márcia Helena Borges, Michael Richardson, Adriano Monteiro de Castro Pimenta, Igor Dimitri Gama Duarte, Maria Elena de Lima

**Affiliations:** 1 Departamento de Bioquímica e Imunologia, Instituto de Ciências Biológicas (ICB), Universidade Federal de Minas Gerais (UFMG), Belo Horizonte, MG, Brasil.; 2 Departamento de Ciências Fisiológicas, Centro de Ciências da Saúde, Universidade Federal do Espirito Santo (UFES), Vitória (ES), Brasil.; 3 Centro de Pesquisa e Desenvolvimento Professor Carlos Diniz, Fundação Ezequiel Dias (FUNED), Belo Horizonte, MG, Brasil.; 4 Departamento de Fisiologia e Farmacologia, Instituto de Ciências Biológicas (ICB), Universidade Federal de Minas Gerais (UFMG), Belo Horizonte, MG, Brasil.; 5 Instituto de Ensino e Pesquisa, Santa Casa de Belo Horizonte, Rua Domingos Vieira, 590, Santa Efigênia, Belo Horizonte, MG, CEP 30.150-240, Brasil.

**Keywords:** spider toxin, Γ-ctenitoxin-Pn1a, PnTx4(5-5), Phoneutria nigriventer, Antinociception, glutamate

## Abstract

**Background:**

The venom of *Phoneutria nigriventer* spider is a source of numerous bioactive substances, including some toxins active in insects. An example is PnTx4(5-5) that shows a high insecticidal activity and no apparent toxicity to mice, although it inhibited NMDA-evoked currents in rat hippocampal neurons. In this work the analgesic activity of PnTx4(5-5) (renamed Γ-ctenitoxin-Pn1a) was investigated.

**Methods:**

The antinociceptive activity was evaluated using the paw pressure test in rats, after hyperalgesia induction with intraplantar injection of carrageenan or prostaglandin E_2_ (PGE_2_).

**Results:**

PnTx4(5-5), subcutaneously injected, was able to reduce the hyperalgesia induced by PGE_2_ in rat paw, demonstrating a systemic effect. PnTx4(5-5) administered in the plantar surface of the paw caused a peripheral and dose-dependent antinociceptive effect on hyperalgesia induced by carrageenan or PGE_2_. The hyperalgesic effect observed in these two pain models was completely reversed with 5 µg of PnTx4(5-5). Intraplantar administration of L-glutamate induced hyperalgesic effect that was significantly reverted by 5 μg of PnTx4(5-5) injection in rat paw.

**Conclusion:**

The antinociceptive effect for PnTx4(5-5) was demonstrated against different rat pain models, i.e. induced by PGE_2_, carrageenan or glutamate. We suggest that the antinociceptive effect of PnTx4(5-5) may be related to an inhibitory activity on the glutamatergic system.

## Background

Accidents provoked by venomous animal are very common, mainly in tropic regions and have long fascinated people. Animal venoms are composed of a great number of substances that are the product of millions of years of evolutionary process. 

In the last four decades, animal venoms have been recognized as potential sources of pharmacological agents and physiological tools. The interest in these venoms has extended into the isolation and the development of new proteins and peptides or their derivatives with therapeutic importance. A good example is Captopril^®^, an antihypertensive drug, which development was based on the pathophysiological studies of *Bothrops jararaca* envenoming, more than a half century ago [1,2]. Another example is ziconotide, a peptide derived from *Conus magus* snail venom, also known under the trade name Prialt^®^, approved by the US Food and Drug Administration in 2004 for the treatment of chronic pains [3,4].

Pain is an unpleasant sensory and emotional experience, subjective and related to multiple mechanisms involved in its generation and transmission [5,6]. Although whole animal venoms are usually related to pain conditions, individual toxins can induce analgesia by selectively inhibiting voltage-gated calcium or sodium channels, ASIC channels or glutamate ionotropic receptors [7]. 

Up to now, many toxins purified from *Phoneutria nigriventer* spider venom have demonstrated their analgesic potential. Among them are some toxins purified from the fraction PhTx3 [8] and PhTx4 [9], which display interesting biological activities in nociceptive models. PnTx3-4 (ω-ctenitoxin-Pn3a), PnTx3-3 (ω-ctenitoxin-Pn2a) and PnTx3-6 (ω-ctenitoxin-Pn4a) are capable of inhibiting voltage-gated calcium channels with different potencies and specificities [10,11,12]. The toxins PnTx3-3 and PnTx3-6 have demonstrated the capacity to control acute and persistent pain states in rodents [13,14]. PnTx3-4 blocks high voltage-activated calcium channels with low specificity [10] and has antinociceptive effect in models of acute and persistent inflammatory and incisional pain in mice [15]. In addition, this toxin was also capable of producing antinociception in the formalin test and this effect was associated with the reduction of glutamate levels in the cerebrospinal fluid. Furthermore, the co-administration of PhTx3-4 and N-methyl-D-aspartic acid (NMDA) dose-dependently reversed the NMDA-induced nociceptive behavior. The antinociceptive effect of this toxin seems to be related to activities in both calcium and glutamate systems [15].

PhTx4 fraction also presents insecticidal activity, because of its high toxicity/lethality to insects (LD_50_ 6.8 ng/20 mg house fly) and very low toxicity to mice (LD_50_ 480 µg/kg mice) [9]. This fraction and its main toxins, PnTx4-3 (δ-ctenitoxin-Pn1b), PnTx4(5-5) (Γ-ctenitoxin-Pn1a) and PnTx4(6-1) (δ-ctenitoxin-Pn1a) inhibit glutamate uptake in cerebrocortical synaptosomes of rats [16,17] and the last two are active on insect sodium channels [18,19,20,21]. PnTx4(5-5), recently renamed Γ-ctenitoxin-Pn1a, is a 47-amino-acid residue polypeptide with a molecular mass of 5.1 kDa. This peptide has a high insecticidal activity in house flies (50 ng/g), cockroaches (250 ng/g) and crickets (150 ng/g). However, when intracerebroventricularly injected in mice (30 µg/mice about 1500 ng/g), little or no behavioral effects are observed [22]. Surprisingly, electrophysiological studies, in rat hippocampal neurons, revealed that PnTx4(5-5) inhibited NMDA-evoked currents, but had little or no effect on the GABA, kainic acid, or AMPA evoked currents [22]. Silva et al. [23] have confirmed that PnTx4(5-5) blocks NMDA receptors in hippocampal slices of mice and hereby had significant neuroprotective effects by reducing glutamate neuronal cell death.

PnTx4(5-5) inhibits the current conducted by NMDA glutamate receptor [22] and glutamate is a key mediator in ascending nociceptive transmission in central and peripheral nervous system [24]. However, after the sting of a prey by a spider, the first targets of the toxins must be in the peripheral nervous system. Moreover, it is not yet known whether this toxin is able to cross the blood-brain barrier. Consequently, it became interesting to study the possible antinociceptive action of PnTx4(5-5) at the peripheral level. This study aimed to investigate the possible antinociceptive effect of PnTx4(5-5) in the peripheral nervous system and proposed an explanation for its mechanism of analgesic action.

## Materials and methods

## Animals

Male Wistar rats weighing 180-200 g were used. They were housed in plastic cages with free access to water and food. Room temperature was maintained at 22 ± 2 °C with a 12h/12h light/dark cycle and (light on from 6:00 a.m). Animals were given at least one day to acclimate to the experimental room before testing. All experiments were performed in accordance with ethical standards and were approved by the Ethics Committee on Animal Experimentation of the Universidade Federal de Minas Gerais (protocol number: 008/2010).

## Drugs and solvents

### *Toxin PnTx4(5-5)*

PnTx4(5-5) has the following sequence: CADINGACKSDCDCCGDSVTCDCYWSDSCKCRESNFKIGMAIRKKFC and it was purified from *P. nigriventer* spider venom, according to the method described by De Figueiredo et al. [22] in the Fundação Ezequiel Dias (FUNED), Belo Horizonte, MG, Brazil. The lyophilized toxin was stored at -20 °C and dissolved in isotonic saline (0.9% NaCl) immediately before use.

### *Hyperalgesic agents*

The hyperalgesic drugs used were carrageenan (Cg - Lamba, Sigma, USA), prostaglandin E_2_ (PGE_2_ - Calbiochem, USA) and L-glutamate (L-Glu - Sigma, USA). The hyperalgesic drugs were dissolved immediately before injections as follows: PGE_2_ (2% ethanol in saline); Cg and L-Glu (saline).

## Nociception measurement

The nociceptive threshold was measured according to the paw pressure test [25]. To perform the measurements, an analgesimeter (Ugo Basile, Italy) was used in which a cone-shaped paw presser with a rounded tip applies a linearly increasing force to the rat’s hind paw. The weight in grams (g) required to elicit the nociceptive response, paw flexion, was determined as the nociceptive threshold, which was considered the average of three consecutive trials recorded. A cut-off value of 300 g was used to prevent damage to the paws.

Hyperalgesia was induced by a intraplantar (ipl) injection of Cg or PGE_2_ or L-Glu into the hind paw of the rats. Considering hyperalgesia as the reduction of the nociceptive threshold, its intensity was evaluated by the difference between the nociceptive threshold recorded at the peak of effect (3 h for Cg and PGE_2_ and 40 minutes for L-Glu), and the value obtained in the beginning of experiments, before any treatment (time zero). The results were expressed in grams (Δ variation of the nociceptive threshold) and graphically represented by bars. In some tests, we evaluated the temporal development of nociceptive threshold of the rats in order to identify the kinetic of the drugs and these results were graphically represented as lines plotted in function of the time.

## Investigation of the peripheral antinociceptive activity of PnTx4(5-5)

Hyperalgesic agents Cg (250 µg) [26] or PGE_2_ (2 µg) [27] or L-Glu (0.5, 1 and 2.5 µg) in a volume of 100 µL were intraplantarly (ipl) injected into the right hind paw of rats. Samples of PnTx4(5-5) (2.5, 5 and 10 µg) in 50 µL/paw were also injected (ipl), 2h30 after Cg and PGE_2_ injection and 5 minutes after L-Glu injection. These times were determined by previous experiments, in which the peptide was administered 30 minutes after Cg and 30, 90 and 150 minutes before Cg injection (data not show). The treatment groups were as follows: controls were injected with the hyperalgesic agents and saline; and experimental groups were injected with the hyperalgesic agent and PnTx4(5-5) in different doses. Each group contained four animals.

To evaluate the local effect of the toxin, the hyperalgesic agents Cg (250 µg) or PGE_2_ (2 µg) or L-Glu (1 µg) were injected (ipl) in a volume of 100 µL into the right and left hind paw of the rats. The toxin PnTx4(5-5) (5 µg against PGE_2_ and L-Glu, and 10 µg against Cg, in a volume of 50 µL/paw) was injected (ipl) in left hind paw of the animals, 2h30 after Cg and PGE_2_ injection and 5 minutes after L-Glu injection. The nociceptive threshold was always measured in the right hind paw of the rats. The control group was injected with only isotonic saline. Each group contained four animals.

## Investigation of the systemic effect of PnTx4(5-5)

The possible systemic action of PnTx4(5-5) against PGE_2_ hyperalgesia was verified by the subcutaneous (sc) administration of 2.5 mg/kg of the toxin, in a volume of 100 µL, in the back of the animals. PGE_2_ (2 µg) was injected (ipl) in the right paw of the rats. The nociceptive threshold was measured always in the right hind paw. The control group was injected with PGE_2_ plus saline and the experimental group was injected with PGE_2_ (ipl) plus PnTx4(5-5) (sc).

## Statistical analysis

The statistical analyses were carried out by one-way and two-way analysis of variance (ANOVA) followed by Bonferroni’s test for multiple comparisons. Probabilities less than 5% (p < 0.05) were considered statistically significant.

## Results

## Systemic effect of PnTx4(5-5) on PGE_2_ induced hyperalgesia

**Figure 1.**
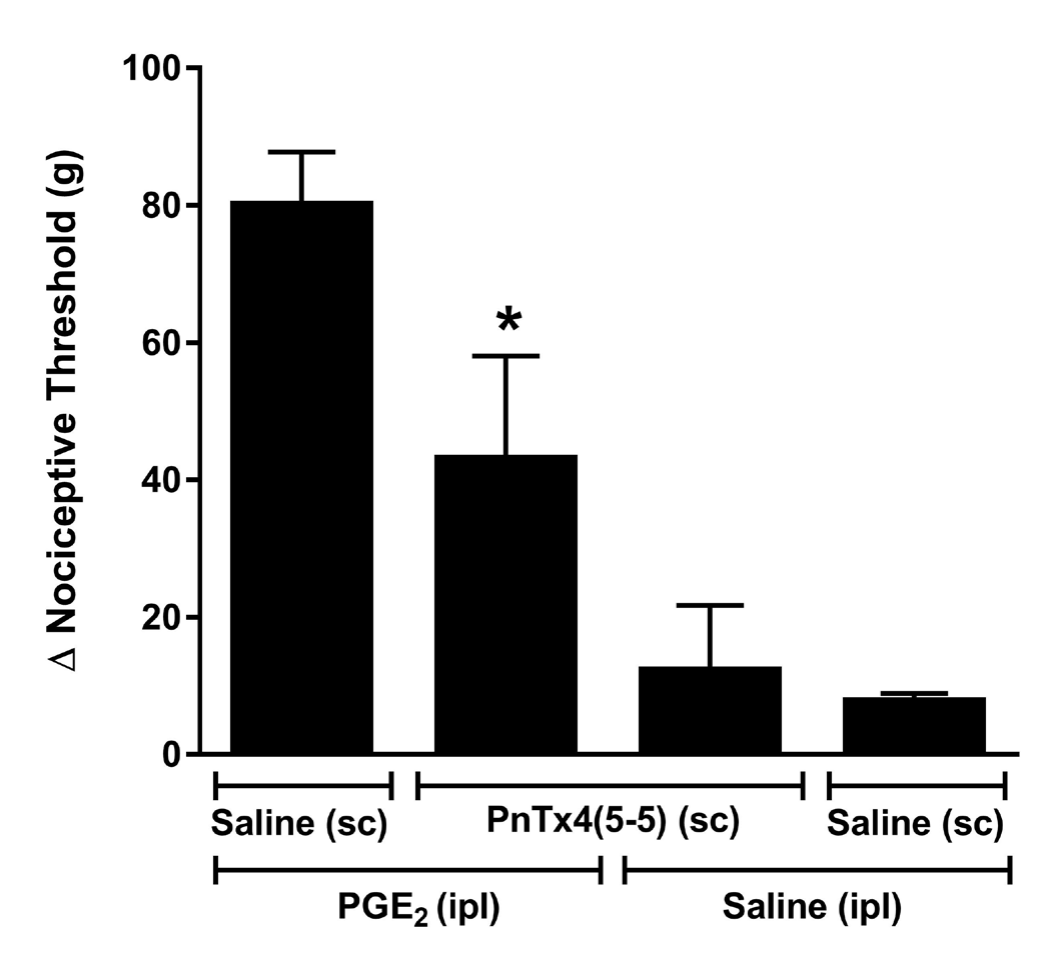
Systemic action of PnTx4(5-5) on PGE_2_ induced hyperalgesia. Effect of PnTx4(5-5) subcutaneously (sc) administrated on the dorsum of rats (2.5 mg/kg) on hyperalgesia induced by PGE_2_ (2 µg/paw). PnTx4(5-5) was administered 150 minutes after intraplantar injection (ipl) of PGE_2_. Each column represents the mean ± S.E.M. of the Δ of nociceptive threshold (g). *Indicates statistical significant difference (p < 0.05) compared to control group (PGE_2_ ipl + saline sc).

In order to evaluate whether the toxin could induce a systemic antinociceptive effect, PnTx4(5-5) (2.5 mg/kg) was subcutaneously administered in the back of the animal, in hyperalgesic state induced by PGE_2_ (2 µg/paw). It was observed that the sc administration of toxin was able to reduce significantly the hyperalgesia induced by PGE_2_ in rat paw, indicating that PnTx4(5-5), in this dose, has a systemic action (Fig. 1).

## Effect of PnTx4(5-5) on carrageenan induced hyperalgesia

**Figure 2.**
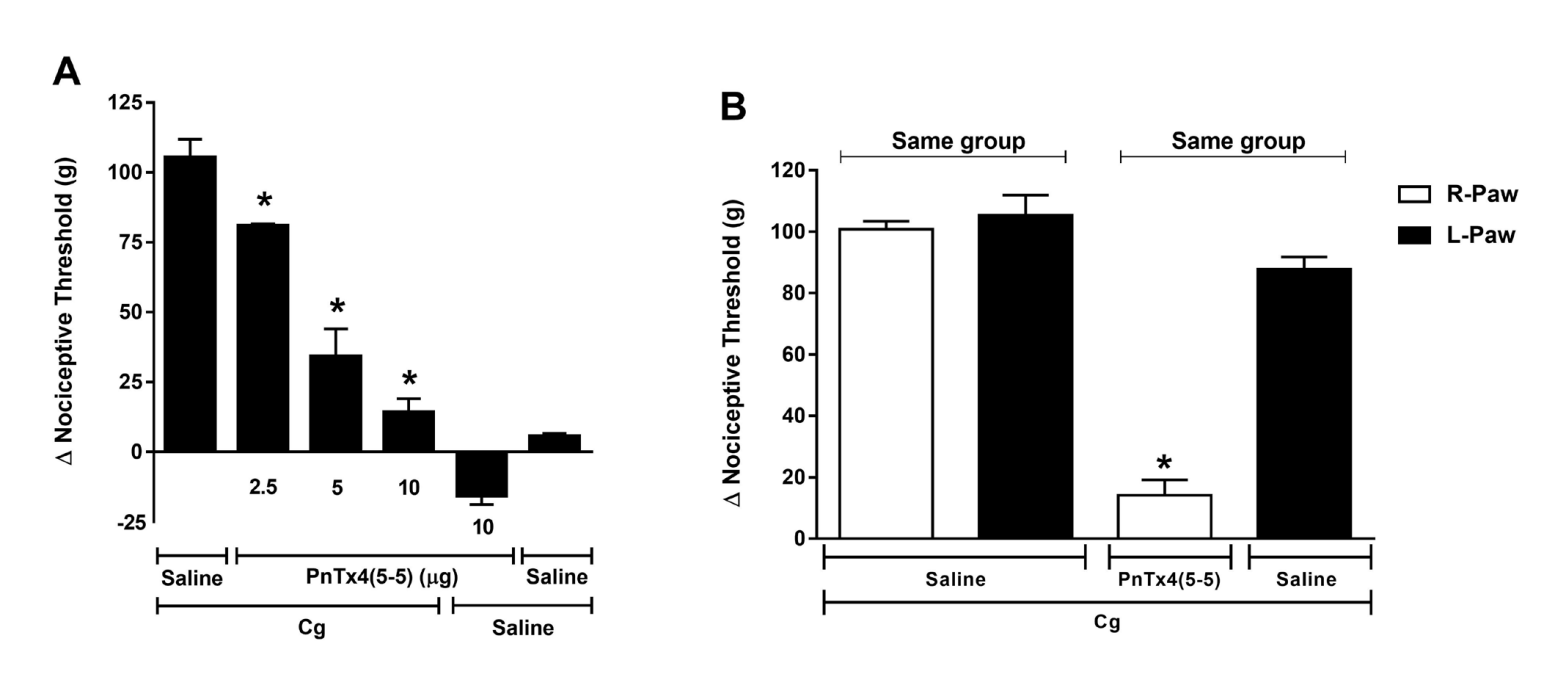
Effect of PnTx4(5-5) on carrageenan induced hyperalgesia. (**A**) Effect of intraplantar administration of different doses of PnTx4(5-5) (2.5, 5 and 10 µg) on hyperalgesia induced by carrageenan (Cg - 250 µg/paw). PnTx4(5-5) was administered 150 minutes after Cg injection. Exclusion of systemic antinociceptive effect of PnTx4(5-5) at a dose of 10 µg. (**B**) Toxin or saline were injected into the right paw (RP) 150 minutes after administration of Cg (250 µg) in both hind paws (RP/LP). Each column represents the mean ± S.E.M. of Δ nociceptive threshold (g) for four animals. *Indicates statistical significant difference (p < 0.05) from the group control (Cg + saline - R-Paw).

Administration of PnTx4(5-5) (ipl) in different doses (2.5, 5 and 10 µg), induced dose-dependent antinociceptive effect following carrageenan‐induced inflammation (Cg, 250 µg/paw). There was a growing decrease of hyperalgesia induced by carrageenan proportionally to the toxin doses (Fig. 2A).

The highest dose tested (10 µg), completely reversed the hyperalgesia induced by Cg, when compared to the control group. Furthermore, the same dose of the toxin, tested without hyperalgesia induction, was not able to cause a significant change in the nociceptive threshold of the animal (Fig. 2A).

PnTx4(5-5) (10 µg) was able to reduce the hyperalgesia induced by Cg when administered on the same paw of Cg (right paw - RP). On the other hand, the toxin was not able to reduce the hyperalgesic state in the contralateral paw (left paw - LP). These data suggest a local action of the toxin against hyperalgesia caused by Cg, at least in this dose (Fig. 2B). 

## Effect of PnTx4(5-5) on PGE_2_ induced hyperalgesia

**Figure 3.**
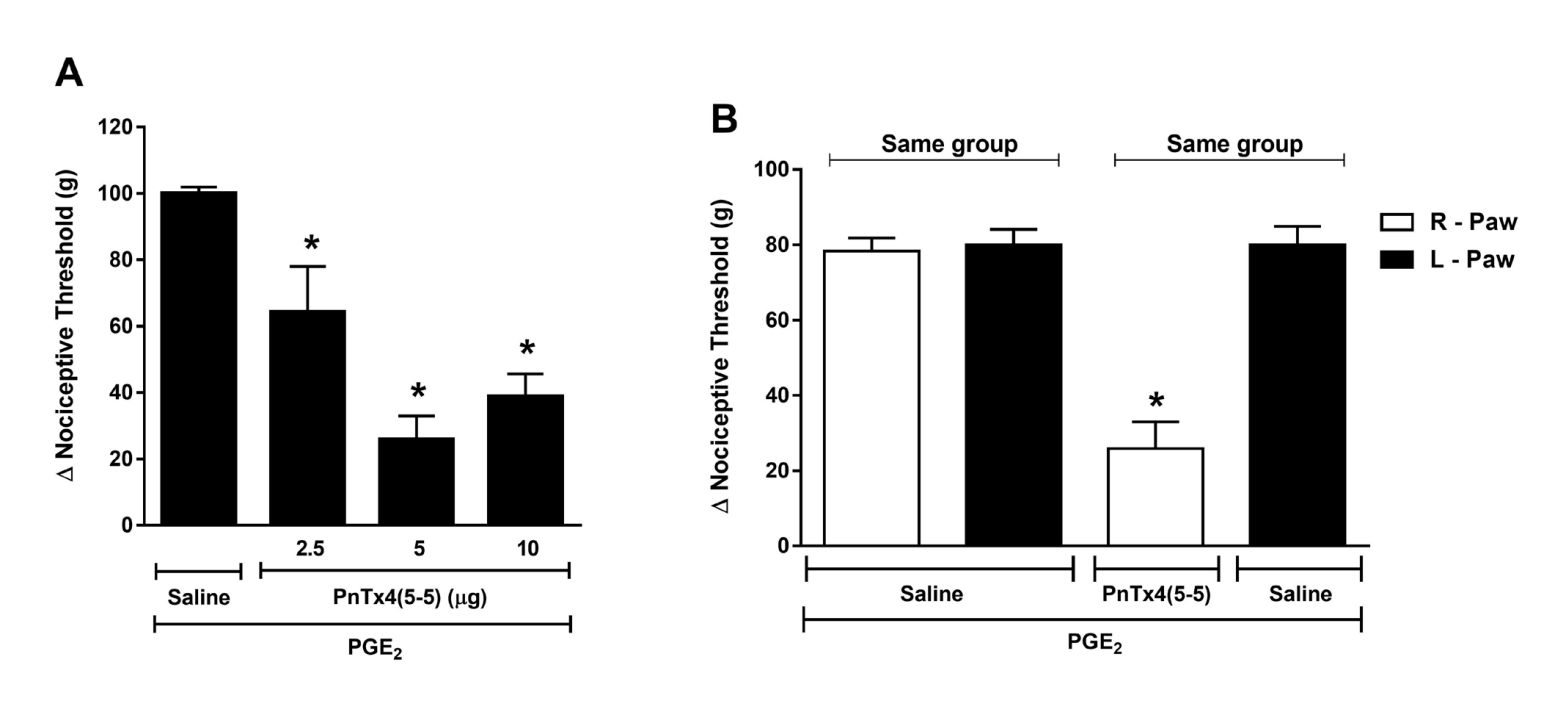
Effect of PnTx4(5-5) on PGE_2_ induced hyperalgesia. (**A**) Effect of intraplantar administration of different doses of the PnTx4(5-5) (2.5, 5 and 10 µg) on hyperalgesia induced by PGE_2_ (2 µg/paw). PnTx4(5-5) was administered 150 minutes after PGE_2_. (**B**) Exclusion of systemic antinociceptive effect of PnTx4(5-5) at a dose of 5 µg. Toxin or saline were injected into the right paw (RP) 150 minutes after administration of PGE_2_ (2 µg) in both hind paws (RP/LP). Each column represents the mean ± S.E.M. of Δ nociceptive threshold (g) for four animals. *Indicates statistical significant difference (p < 0.05) from the group control (PGE_2_ + saline - R-Paw).

To evaluate the antinociceptive effect of PnTx4(5-5) against the hyperalgesia induced by PGE_2_, doses (2.5, 5 and 10 μg) were administered via ipl, 150 minutes after the hyperalgesia induced by PGE_2_ (2 µg/paw). Figure 3A shows the antinociceptive effect of PnTx4(5-5) under the hyperalgesia induced by PGE_2_. The doses 2.5, 5 and 10 µg of the toxin were able to reverse the nociceptive threshold reduced by PGE_2_. There is no statistical difference between the antinociceptive effect induced by the doses of 10 and 5 µg (Fig. 3A).

The antinociceptive local effect of PnTx4(5-5) was performed, as in the inflammatory model (Cg), under PGE_2_ hyperalgesia and was shown that the lower dose with maximal effect (5 µg) did not reduce the hyperalgesia induced in the contralateral paw (left paw - LP) (Fig. 3B).

## Hyperalgesic effect of L-glutamate (L-Glu)

**Figure 4.**
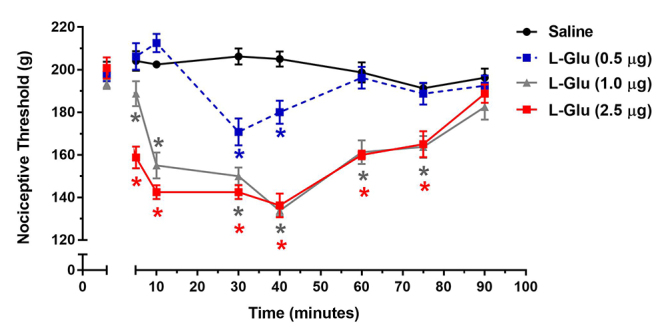
Temporal development of hyperalgesia induced by intraplantar injection of different doses of L-glutamate (L-Glu). L-Glu was administered via intraplantar in the doses of 0.5, 1.0 and 2.5 µg. Each symbol represents the mean ± S.E.M. of Δ of nociceptive threshold (g) for four animals. *Indicates statistical significant difference (p < 0.05) from the control group (saline).

It is well known that the administration of glutamate can promote hyperalgesia. In a very interesting work, Gazerani et al. [28] demonstrated for the first time that subcutaneous injection of glutamate evokes pain, vasomotor responses and hyperalgesia in humans. As expected, in our experiments, the intraplantar administration of L-glutamate induced a dose-dependent decrease of the nociceptive threshold of the rats, when compared to the control group (saline) (Fig. 4). In this assay, the hyperalgesic effect can be observed five minutes after the injection of the doses of 1.0 and 2.5 µg, and the maximum effect was observed in 30-40 minutes after L-Glu administration. The hyperalgesia induced by L-Glu in the highest dose tested (2.5 µg) was not statistically different from the dose of 1.0 µg; therefore the dose of the 1.0 µg was chosen to perform the subsequent experiments.

## Effect of PnTx4(5-5) on hyperalgesia induced by L-glutamate (L-Glu)

It was previously shown that the toxin PnTx4(5-5) can inhibit NMDA glutamate receptor currents in hippocampal neurons of rats [22]. Thus, to evaluate the analgesic effect of PnTx4(5-5) in the hyperalgesia induced by L-Glu, the toxin was administered via ipl (5 µg) five minutes after the hyperalgesic induction by L-Glu (1 µg/paw) and measured at 40 minutes. 

**Figure 5.**
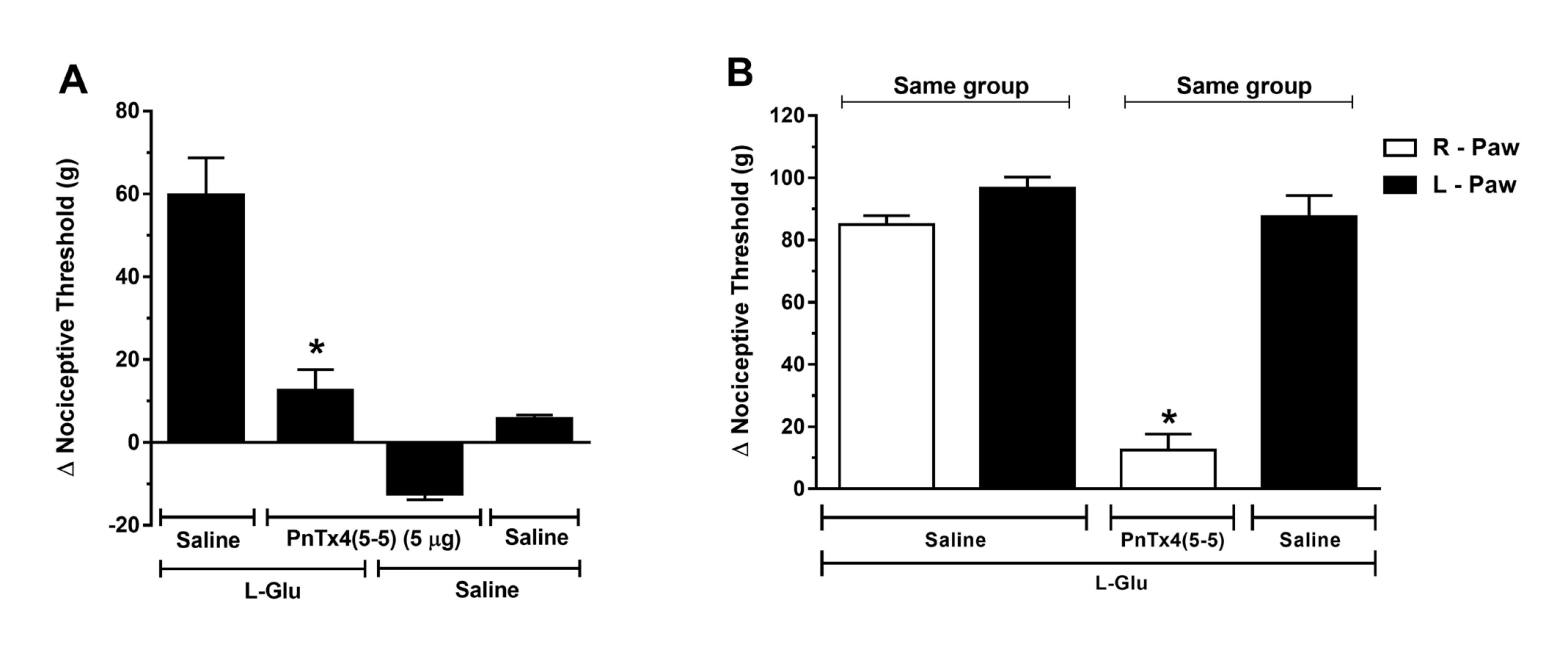
Effect of PnTx4(5-5) on L-Glu induced hyperalgesia. (**A**) Effect of intraplantar administration of PnTx4(5-5) (5 µg) on hyperalgesia induced by L-Glu (1 µg/paw). PnTx4(5-5) was administered 5 minutes after L-Glu. (**B**) Exclusion of systemic antinociceptive effect of PnTx4(5-5) at a dose of 5 µg. Toxin or saline were injected into the right paw (RP) 5 minutes after administration of L-Glu (1 µg) in both hind paws (RP/LP). Each column represents the mean ± S.E.M. of Δ nociceptive threshold (g) for four animals. *Indicates statistical significant difference (p < 0.05) from the control group (L-Glu + saline - R-Paw).

The results (Fig. 5A) show that PnTx4(5-5) induced a significant antinociception when compared to the control, L-glu + saline. The capacity of PnTx4(5-5) (5 µg) to neutralize the hyperalgesia caused by L-Glu was similar to the one observed for the hyperalgesia induced by Cg and PGE_2_. Furthermore, 5 µg of the toxin did not reduce hyperalgesia in the contralateral paw (Fig. 5B).

## Discussion

The present investigation evaluated the antinociceptive potential of PnTx4(5-5), a toxin purified from the venom of the “armed” spider *P. nigriventer*. Our results showed the analgesic effect of PnTx4(5-5) in inflammatory and nociceptive pain models and revealed the possible involvement of the glutamatergic system in this effect.

The first nociceptive pain model employed was the inflammatory one, induced by intraplantar administration of carrageenan. When tested in the periphery, PnTx4(5-5) promoted a dose-dependent antinociceptive effect (Fig. 2A). In this pain model, a kinetic of release of several inflammatory mediators occurs, including histamine, serotonin, kinins and prostaglandins, which are known to activate nociceptors and to decrease the threshold of nociceptors activation [29,30]. The kinetics of mediators release in carrageenan inflammation may explain why PnTx4(5-5) has its analgesic effect only when intraplantarly administered 2 h and 30 min after carrageenan injection (data not show).

Similar results were found for PnTx4(6-1) (δ-ctenitoxin-Pn1a), when tested in the same hyperalgesic conditions [31]. These two homologous toxins, purified from *P. nigriventer* venom, share 64% of identity including ten conserved cysteine residues [22]. These ten cysteine residues may be important for the tertiary structure of these toxins and it can be assumed that PnTx4(6-1) and PnTx4(5-5) have a very similar structural conformation, that could be important to the analgesic activity.

The second model used was the nociceptive pain model, obtained by the intraplantar injection of prostaglandin E_2_ (PGE_2_) into the paw of the rats. When administered in the same place, PnTx4(5-5) (25 µg/kg) showed an antinociceptive effect in PGE_2_-induced hyperalgesia, in this dose the toxin has a local effect (Fig. 2). It was also shown that the subcutaneous administration of the toxin, in the animal’s back, was able to reduce significantly the hyperalgesia induced by PGE_2_ in the rat paw, indicating that PnTx4(5-5), in a dose a hundred times higher (2.5 mg/kg), has a systemic action (Fig. 1).

PGE_2_ is released after induction of cyclooxygenase-2 in injured tissues and cells, binding in their specific receptors. PGE_2_ promotes activation of intracellular signaling pathways that in the end leads to phosphorylation of a number of target proteins including TRPV1 channels, T-type calcium or voltage-gated Na^+^ channels, which contribute to sensitization mechanisms in the peripheral nervous system [32]. 

It is well known that voltage-gated sodium channels (Nav) are responsible for the initiation of action potential in neurons, including the neurons involved in pain process. A variety of sodium channels are expressed in somatosensory neurons, including the tetrodotoxin (TTX)-sensitive channels Nav1.1-Nav1.4, Nav1.6 and Nav1.7, and the TTX-insensitive channels, Nav1.5, Nav1.8 and Nav1.9 [33]. Recently, PnTx4(5-5) was cloned and heterologously expressed in *Escherichia coli*. This recombinant toxin [rPnTx4(5-5)] potently inhibited the inactivation of insect sodium channels likely to the native toxin. In addition, when tested on mammalian Navs (Nav1.2 to Nav1.6), expressed in *Xenopus leavis* oocytes, rPnTx4(5-5) differentially inhibited the sodium current peak amplitude of all the mammalian channels tested, with the highest current inhibition for Nav1.3 (38.43 % ± 8.04 %, IC50 = 1.5 μM) [21]. 

In order to compare the recombinant and the native toxin Paiva et al. [21] tested both toxins in Nav1.3 and Nav1.5. The percentage of maximal inhibition at 1 μM on Nav1.3 was 38.4 ± 8.1% and 36.7 ± 6.3%, and on Nav1.5 it was 18.3 ± 1.6% and 20.3 ± 1.2% for the recombinant and native toxin, respectively. Similarly to the recombinant toxin, the native one can inhibit sodium currents and this inhibition could help to explain the antinociceptive effect observed for PnTx4(5-5). Interestingly, among the tested channels, the inhibitory effect of the recombinant toxin rPnTx4(5-5) was higher in Nav1.3 and Nav1.6 channels subtypes, being the effect in the last comparable for the native toxin [21]. It is known that Nav1.3 expression is high during development but nearly undetectable in adulthood [34]. Nevertheless, upon nerve injury and demyelination, Nav1.3 expression is strongly upregulated [35,36]. On the other hand, Nav1.6 channels are related to neuropathic pain states, including thermal pain [35,37]. Nav1.6 channels are densely expressed in nodes of Ranvier in myelinated sensory afferents and the expression of these channels increases in these nodes with nerve damage [38]. Nav1.6 channels generate resurgent currents that facilitate repetitive firing contributing to persistent pain sensation [39]. These activities suggest that PnTx4(5-5) could be a good candidate as drug model to treat pain in these injuries. However, its activity in other channels, i.e Nav1.5, although lower than that in Nav1.6 and Nav1.3, must be taken in account.

In Table 1 we bring a brief comparison among the effective doses to induce antinociception of PnTx4(5-5) and other commercial analgesic molecules. All molecules are tested in carrageenan or PGE_2_ pain models and measures were performed by using the same paw pressure test [25]. It is noteworthy that among all the tested drugs, PnTx4(5-5) in general showed higher activity. However, the poor knowledge of the mechanism of action of PnTx4(5-5) in nociception, as well as the different mechanisms of these drugs make a more accurate comparison difficult.

**Table 1.** Comparative table of doses of substances that induce antinociceptive effect when administered in rat paw

Toxin/drug	Dose (µg/paw)	Reference
PnTx4(5-5)	2.5 to 10	-
Dipyrone	200	[40]
Diclofenac	100	[27]
Indomethacin	200	[27]
Morphine	100	[41]
Fentanyl	1.5	[42]

Glutamate is the major mediator of excitatory signals in the central nervous system of mammals and is involved in physiologic and pathologic processes, such as excitatory synaptic transmission, synaptic plasticity, cell death, stroke, and chronic pain [33,43]. 

Recent work has shown that PnTx4(5-5) blocks NMDA receptors in hippocampal slices, which is associated with neuronal cell death decrease in two important neurodegenerative disease models [23]. PnTx4(5-5) has a potential to be neuroprotective to corticostriatal neurons from mouse model of Huntington’s disease and was efficient to protect neurons from Aβ-induced insult (Aβ is the main component of Alzheimer’s disease amyloid plaques) [23]. In our work, we suggest that the antinociceptive effect of PnTx4(5-5) can also be related to the glutamatergic system, since the toxin was able to reverse de hyperalgesia induced by intraplantar administration of L-glutamate. In 1992, it was demonstrated that the intrathecal administration of a NMDA antagonist (MK-801) dose-dependently reduced carrageenan-induced thermal hyperalgesia in rats [44]. More recently, it was reported that, besides of inducing pain behavior, carrageenan injected in the paw altered GluA1 and GluA4 trafficking in the plasma membrane in the neurons of dorsal horn. These effects were mediated by spinal TNF release through PI3K pathway with involvement of the NMDA pathway [45]. Combined, these results indicate a very close relation between peripheral inflammation and NMDA receptors.

Another antinociceptive toxin isolated from *P. nigriventer* venom, PnTx3-4 (ω-ctenitoxin-Pn3a), seems to correlate the glutamate system and analgesia [15,46]. In addition to the effect in calcium channels, PnTx3-4 caused a remarkable reduction of dead cells in the retina layers in NMDA-induced injury, and a high neuroprotective effect in the ganglion cell layer. This toxin also reduced glutamate excitotoxicity, reactive oxygen species production and oxidative stress, all pathophysiological processes involved in retinal injury [47]. In a previous work, the antinociceptive effect of this toxin in inflammatory and incisional pain models was shown and this effect seems to be related to both calcium and glutamate systems [15].

## Conclusion

PnTx4(5-5) has an analgesic effect in inflammatory and nociceptive pain models and considering its already demonstrated activity in Nav1.3 and Nav1.6 channels, it could be an effective drug model to treat pain in diseases involving nerve damage. However, further studies are still necessary to better clarify the mechanism involved in the nociceptive effects of PnTx4(5-5).

### Abbreviations

 Cg: carrageenan; ipl: intraplantarly; L-Glu: L-glutamate; LP: left paw; Nav: voltage-gated sodium channels; NMDA: N-methyl-D-aspartic acid; PGE_2_: prostaglandin E_2_; RP: right paw; Sc: subcutaneously.
